# Implementation and Evaluation of a RFID Smart Cabinet to Improve Traceability and the Efficient Consumption of High Cost Medical Supplies in a Large Hospital

**DOI:** 10.1007/s10916-019-1269-6

**Published:** 2019-05-10

**Authors:** María del Carmen León-Araujo, Elisa Gómez-Inhiesto, María Teresa Acaiturri-Ayesta

**Affiliations:** 1 Purchasing and Repository Department, Cruces University Hospital, Barakaldo, Spain; 2 Departamento de Compras y Almacén, Hospital Universitario Cruces, Plaza de Cruces n° 12, 48903 Barakaldo, Bizkaia Spain

**Keywords:** RFID, High value product, Surgery, Traceability, Logistics

## Abstract

The efficiency of a smart cabinet with RFID technology to improve the information about inventory management for cardiothoracic surgery as well as for time savings, was assessed in a large reference hospital. In a 6-month study, the implemented operational RFID process (StocKey® Smart Cabinet) consisted of: i) product reception, registration and labelling in the general warehouse; ii) product storage in the cabinet and registered as inputs by radiofrequency; iii) products registered as outputs as required for surgery; iv) product assignment to a patient in the operating room; and v) return of products not used to the cabinet. Stock-outs, stock mismatches, urgent restocking, assignment of high-value medical products to patients, and time allocated by the supervisory staff to the stock management, were assessed on a monthly basis. 0% stock-outs and 0% stock mismatches using RFID were observed during the study. Monthly percentages of products requiring urgent restocking ranged from 0% to 13.3%. No incorrect assignments to patients of surgery products or prostheses were detected. The percentage of correct assignments increased from 36.1%–86.1% to 100% in the first 4–5 months. The total average time allocated by the supervisory staff to the whole logistic chain was reduced by 58% (995 min with the traditional manual system vs. 428 min with RFID). The RFID system showed the ability to monitor both the traceability and consumption per patient of high-value surgery products as well as contributed to significant time savings.

## Introduction

Traditionally, all activities related to hospital logistics have been aimed at providing the health care staff with the necessary supplies to cover patient needs no matter the situation [26]. It is estimated that logistics represent up to 45% of a hospital’s operational budget [7]. Nowadays, the perception that the patient can also be considered a client is gaining acceptance, so that health care centers are encouraged to develop sustainable management models in order to improve their efficiency, speed and reduce waste [8, 17]. Therefore the adoption of novel technologies to account for the right stock, timely replenishment cycles, traceable flow of items and adequate consumption per patient is warranted. Such developments should ultimately have a positive impact on patient safety.

The automation of logistic systems enables progress towards efficient per patient management models, as it streamlines tasks associated with managing the full traceability of materials and obtaining information on consumption per patient that, without this automation, would be nearly impossible given the large number of resources and staff hours that would be required. Automatic identification technologies such as barcodes and more recently radio-frequency identification (RFID) have been developed to manage traceability efficiently. In contrast to barcode technology, which is still subject to operational problems related to manual processes [9], RFID technology offers accurate inventory visibility in real time, and immediate identification of the exact location of any individual item [2]. In addition, RFID-enabled cabinets can be used in the replenishment of consignment and high value supplies, such as in operating rooms [3]. Although RFID cabinets to track medical supplies have been in use since the early 2000s [24], studies evaluating their efficiency in the perioperative environment are scarce.

The University Hospital Cruces in Barakaldo (Spain), integrated into the Basque Health Service, has 981 beds and it is a regional reference hospital with services in complex specialties such as pediatric cardiac surgery, kidney and liver transplantation, and major burns. The hospital logistics manages approximately 15,000 stock references, of which around 4150 correspond to implantable devices and prostheses. Because of the high cost of these products, ensuring their traceability is of critical importance [1].

Recently the University Hospital Cruces has been investing in technologies for the improvement of logistics management through the automation of tasks and processes. Different types of automation equipment within all of the warehouses have been implemented. Thus, the peripheral warehouses (those with automated permanent inventory, located in the hospital Services and Departments) have successfully implemented the StocKey® Kanban “double bin” system with radiofrequency identification (Grifols, Barcelona, Spain), that allows inventory management (e.g., stock levels, batch, expiration dates) [22]. Taking a step further, the StocKey® RFID Smart Cabinet (Grifols) that provides access control for tracking high value products is being implemented to complement the Kanban system [15].

In this study, the efficiency of the RFID for the monitoring of traceability and consumption of high value surgical supplies was assessed in the cardiothoracic surgery operating rooms of the Hospital Cruces.

## Materials and methods

### Objectives

The primary objective of this study was to assess the improvement in the information about inventory management for cardiothoracic surgeries associated with the implementation of a simple and user-friendly automated system (StocKey® RFID Smart Cabinet) for the traceability and monitoring of medical supplies and products.

Specific endpoints included assessing the product traceability and consumption per patient of surgery supplies, control of lots, stocks and expiration dates of surgery supplies, as well as switching from paper-based records to electronic sheets. Finally, time savings in the supervisory staff derived from the RFID implementation were evaluated in comparison to the former non-automated logistics system of the Hospital Cruces.

### Description of the RFID system

**Fig. 1:**
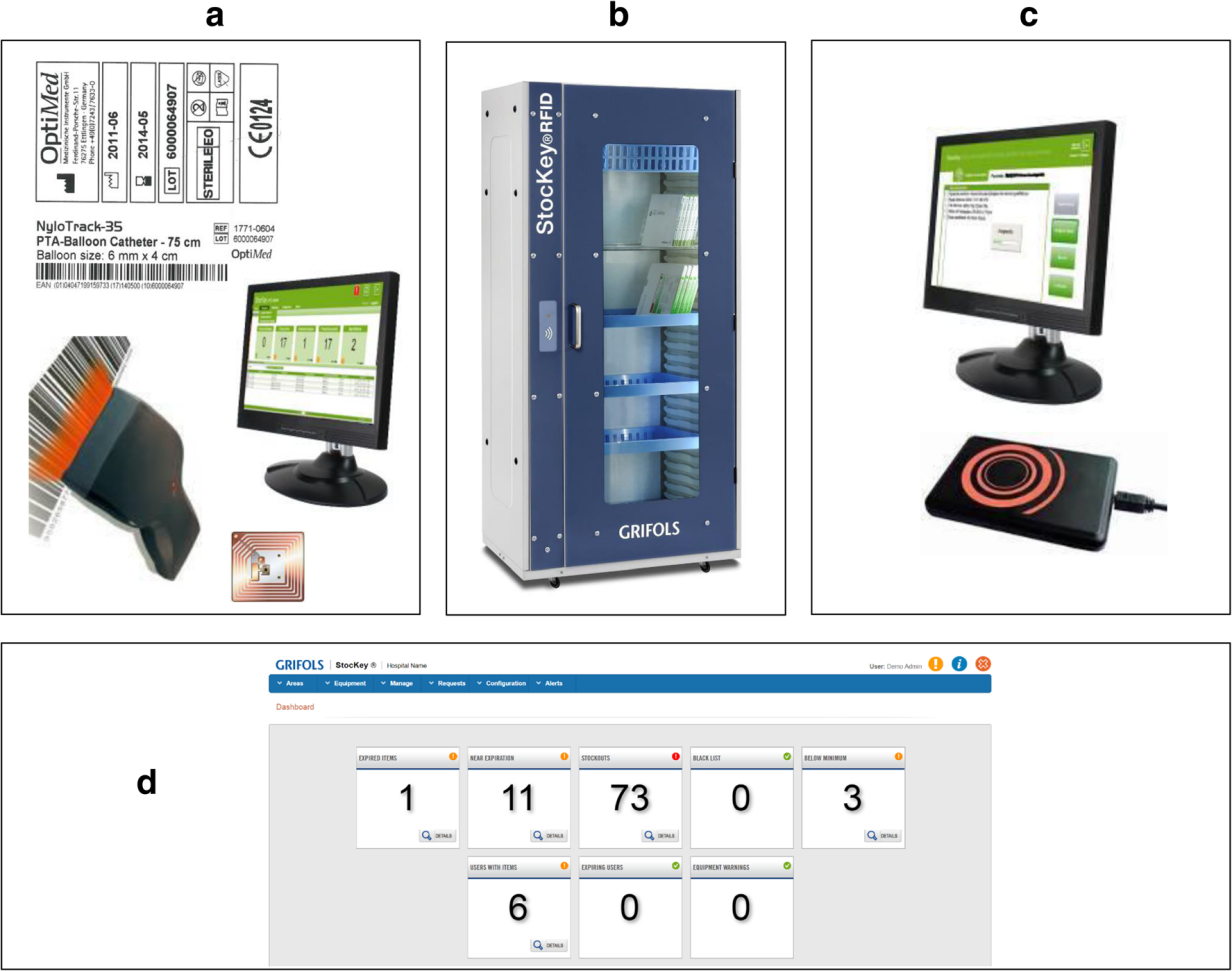
The RFID Smart Cabinet components: A) labeling station; B) Closed cabinet; C) Patient assignment station; D) Dashboard with KPI’s (Key Performance Indicators) and integrations with the Hospital Informatics System

The StocKey® RFID Smart Cabinet consists of: i) a labeling station, that recognizes the information of the GS1 barcodes (format EAN & HIBC), for the assignment of RFID labels with all information for the identification of the product item (e,g,. batch, serial number, expiration date); ii) a closed cabinet (Faraday cage with 816 L capacity in the large version) where products are stored (with a transparent door and lights inside to see products; flexible internal configuration of bins, shelves, dividers, hangers, etc.), secured with access controlled by a proximity card; all inputs and outputs are read by radiofrequency and send stock data when closing the door, as well as assigned to a user (nurse); iii) a patient assignment station in the operating room (with touchscreen, card reader and RFID reader) to check items assigned to a user and assign items to a patient by reading RFID tags, during the intervention; and iv) centralized software with web access for user management, integrations with the Hospital Informatics System and dashboard with KPI’s (Key Performance Indicators) such as stock control and system alerts (expired products; products with early expiration date; products under minimal stock; stock-outs; products with medical alerts such as a recall; and users with items unassigned to patient for more than 48 h). The components of the StocKey® RFID Smart Cabinet are illustrated in Fig. 1.

The operational process from reception to assignment to patient and accounting is as follows:

1. The products are received in the general warehouse; the RFID labels are applied to each item and registered into the system through RFID and barcode reading.
2. Tagged products are stored in the RFID cabinet by the logistics staff who have the access card. As being registered as inputs by radiofrequency, the products are incorporated into the electronic inventory of the cabinet.
3. As a stored product is required for surgery, a nurse with the access card removes the item from the RFID cabinet, and both the user and item output are registered.
4. In the operating room, the item is assigned to a patient through the RFID reader at the patient station. The patient has been previously tagged and identified through hospital healthcare information and surgical programming systems of the Basque Health Service. If patient assignment was not performed on the day of the intervention, the RFID system posted the product as “not assigned”.
5. The items not used in the intervention are returned to the RFID cabinet and automatically re-integrated into the electronic inventory without the need for manual registration.
6. The cost of the items used is automatically accounted for through integration with the economic management system of the hospital. The consumed items are restocked in the RFID cabinet on a daily basis.
7. Additionally, the products assigned can be ascribed to the clinical records of the patient via software integrations, as well as sharing the stock needs with the suppliers to allow for a quicker restocking.

### Implementation of the RFID system in the hospital

The startup of the RFID was preceded by a pre-market pilot test carried out at the Hospital Universitario Cruces in a real world environment of an operating room.

In the implementation of the RFID, both the different actions to be taken as well as the timings were considered. The process took place from March 2014 to July 2015 and the most relevant steps are summarized as follows:

- Set up of an implementation timetable and selection of personnel.
- Validation of the RFID system in a test environment. This included the training of users, data registration, and integration of the system with the economic management and information systems of the hospital and the Basque Health Service.
- Definition and selection of high cost medical products for special monitoring by RFID: prostheses, implants, staplers, laparoscopy items, stents, catheters, valves.
- Parameterization of the variables to be RFID-monitored, with a full record of relevant data having an impact on the clinical safety of the patient: batch, serial number, expiration date, supplier, reference.
- Consideration of the appropriate places to install the RFID stations and installation of the RFID cabinets in the operating rooms.

Based on the experience acquired during the pilot test, areas for improvement detected through the RFID implementation were either applied immediately (such as those aimed at a more convenient and simpler use of the tool by the staff members as well as those involved in integration processes) or programmed for future developments.

### Evaluation of the RFID system

From July to December 2015, the efficiency of the RFID for product traceability and assignment to patient (consumption) was tested in the cardiothoracic surgery operating rooms. The following variables were defined and calculated on a monthly basis:

- Medical products correctly assigned to patients: number of items assigned to patients with respect to total items consumed during an intervention.
- Prostheses correctly assigned to patients: number of prostheses assigned to patients with respect to total prostheses consumed during an intervention.
- Urgent restocking: number of products for urgent restocking (too few items in stock) with respect to the number of products requested.
- Stock mismatches: number of mismatches between products in the cabinet and RFID system stock with respect to the total number of products.
- Stock-outs: number of products not in stock with respect to the total number of products.

The monthly objectives to be reached were: i) 100% of correct assignments of surgery medical items and prostheses: ii) 0% of stock-outs and stock mismatches; and iii) no more than 15% of urgent replacements. Since the former non-automated system did not generate data to design a comparative study, the stock-outs standard of the Hospital Cruces for healthcare supplies (1%) was taken as an indicative figure, and the quality indicators for technologies applied to the outpatient hospital pharmacy as per the Spanish Society of Hospital Pharmacy [10] were considered as guidance.

In addition, the average monthly time allocated by the supervisory staff to the stock management during the study period (requests of products for cabinet, requests of other products, forms filling, receiving of products, product review, stock-outs) was calculated and compared to the equivalent period before the implementation of the RFID system.

## Results

### RFID system set-up

A total of 83 users were authorized to the RFID system, including supervisors and administrators with full access, warehouse workers with access to the labelling station, ancillary workers with access to the cabinet for restocking, and operating room nurses with access to the cabinet and patient assignment station. A total stock of 250 tagged items was set out in the RFID cabinet, of which 72 were surgery materials such as mechanical suture, sealing and cutting, while 178 were implantable materials (prostheses, implants, staplers, stents, catheters, heart valves). The estimated cost of the stored stock was € 369,211.

### Product traceability and consumption results

During the 6-month study period, the number of surgery products and prostheses consumed were 425 and 249, respectively.

Results of traceability variables related to stocks during the study period are shown in Table 1. The objective of 0% stock-outs and 0% stock mismatches was achieved throughout the study period. The percentage of products requiring urgent restocking was within the objective set, ranging from optimal 0% in November to 13.3% in December.

**Table 1:** Results of traceability variables related to stocks as registered by the RFID during the study period

	July n (%)	August n (%)	September n (%)	October n (%)	November n (%)	December n (%)
Urgent restocking	2 (11.8)	1 (6.3)	1 (4.8)	3 (13.0)	0 (0)	2 (13.3)
Total requests	17	16	21	23	17	15
Stock-outs	0 (0)	0 (0)	0 (0)	0 (0)	0 (0)	0 (0)
Stock mismatches	0 (0)	0 (0)	0 (0)	0 (0)	0 (0)	0 (0)
Total stock	250	250	250	250	250	250

No incorrect assignments of surgery items or prostheses to patients were detected. The correct assignment of surgery items to patients increased from 36.1% to 100% in November while the correct assignment of prostheses to patients increased from 86.1% to 100% in October. During the subsequent months, the percentage of correct assignments slightly decreased. These results are detailed in Table 2. The reasons for the few cases of unsuccessful assignment of the product to patient were essentially of a technical nature and data integration.

**Table 2:** Results of the assignments of surgery products and prostheses to patients by the RFID during the study period

		July n (%)	August n (%)	September n (%)	October n (%)	November n (%)	December n (%)
Assignment of surgery products to patient	Correctly	22 (36.1)	51 (63.8)	65 (92.9)	68 (82.9)	70 (100)	52 (83.9)
	Incorrectly	0 (0)	0 (0)	0 (0)	0 (0)	0 (0)	0 (0)
	Not assigned	39 (63.9)	29 (36.3)	5 (7.1)	14 (17.1)	0 (0)	10 (16.1)
	Total	61	80	70	82	70	62
Assignment of prostheses to patient	Correctly	31 (86.1)	17 (85.0)	29 (93.5)	51 (100)	45 (93.8)	40 (88.9)
	Incorrectly	0 (0)	0 (0)	0 (0)	0 (0)	0 (0)	0 (0)
	Not assigned	5 (13.9)	3 (15.0)	2 (6.5)	0 (0)	3 (6.2)	5 (11.1)
	Total	36	20	31	51	48	45

### Time allocation results

The total average time allocated by the supervisory staff to the whole logistic chain was reduced by 58% (from 995 min with the traditional manual system to 428 min with the RFID system). The task most dramatically reduced was product request by the supervisory staff (100% reduction due to full automation), followed by product receiving by the supervisory staff (56% reduction due to transfer of allocation to the logistics staff). Times of the other monitored tasks were not affected by the RFID system. These results are summarized in Table 3.

**Table 3:** Total average time allocated to the whole logistic chain, before (manual system) and after the implementation of the RFID

Task	Manual system		With RFID	
	Responsible	Allocation time (min)	Responsible	Allocation time (min)
Product request for cabinet	Supervisor	400	Automated	0
Requests of other products	Supervisor	20	Supervisor	20
Forms filling	Supervisor	220	Supervisor	220
Products receiving	Supervisor	300	Logistics	133
Product review	Supervisor	15	Supervisor	15
Stock-outs	Supervisor	35	Supervisor	35
Other tasks	Supervisor	5	Supervisor	5
Total		995		428

## Discussion

In the context of economic adjustments and resource optimization in global healthcare without jeopardizing patient safety, reliable systems to monitor the consumption of materials through ensuring their full traceability are needed [13, 28]. The RFID automation of logistic systems represents a significant step forward in patient-focused models for consignment and high value product management [4]. Benefits of RFID implementation address patient safety issues (e.g., preventing the implant of a recalled or outdated product or a product stock-out during the surgical intervention), healthcare providers (e.g., fewer manual processes and non-clinical tasks), and financial status (e.g., preventing product wastage due to expiration or obsolescence, excess of stock or stock diversion) [14, 16, 25].

In this study, the RFID system demonstrated not only having the capacity for full traceability and monitoring of high value medical products in the perioperative cardiothoracic surgery, but also the ability to significantly reduce the time that the clinical staff allocates to the tasks of the logistic chain.

RFID technologies in hospital logistics have been commonly applied in management of pharmaceutical inventories [6], traceability of medication distribution from warehouse to pharmacy [18, 23], and from pharmacy to the patient [12, 19–21]. With a scarcity of empirical studies on RFID use for stock management of high value surgery medical products, our results would support the implementation of a RFID smart cabinet for this aim. The RFID was proven highly efficient for the control of stocks during the study period, since no stock-outs and no stock mismatches were observed. The products were fully traceable from their reception in the general warehouse, to assignment to surgery patient, or re-integration into the electronic inventory if not used. In addition, the system effectively recorded the consumption of products per surgery and per patient.

Management of critical products through RFID was aimed to achieve a minimal percentage of urgent re-stocking. Our results showed a reasonable range of 0% to 13.3% values, although there was a margin for improvement to optimal 0% throughout considering that the RFID implementation project is in the very early stages. It should be taken into account that unexpected shortages of surgical items may have a severe impact in patient safety.

In the operating room, no incorrect assignment of surgery products and prostheses to patients were observed during the study period, which highlights the contribution of the RFID system to patient safety. The percentage of correct assignments increased from 36.1%–86.1% to 100% in the first 4–5 months, which represented good progression. The remaining percentage of not assigned products during some months was essentially due to technical reasons and data integration. Further corrective action was applied as programmed in the RFID implementation program. Nevertheless, the observed results fell within what could be expected at the very early stages of the implementation project.

In addition to improved product traceability and increased patient safety, better staff time management was one of the focuses of RFID implementation. Nurse activities such as product administration and documentation represent targets for improving efficiency [11]. Our study demonstrated a significant reduction of time allocation of the supervisory staff nurses after the implementation of the RFID. Importantly, this time was mainly saved from administrative and warehouse tasks. Increasing the efficiency of nursing care delivery is essential to hospital function and patient safety.

RFID implementation in surgery is not without risks. In addition to organizational issues as we encountered, environmental (e.g., interference with radio waves and electromagnetic fields, inhibition of temporary pacing systems) and technical (e.g., reading errors; breakdown of computer networks) hazards also need to be considered when adopting RFID in surgery facilities [5, 27]. In the case of StocKey® RFID, it is a Faraday cage that shields their contents from electronic interferences. Finally, our study was limited to our hospital and three operating rooms, so extrapolation of conclusions should be made with caution.

In summary, the RFID smart cabinet tool for inventory management demonstrated its capacity for the traceability and monitoring of high-value medical products stock, from warehouse to patient assignment in the cardiothoracic operating room. Moreover, the RFID smart cabinet significantly reduced the time allocated to the logistic chain tasks by the clinical staff. We believe that the implementation of a RFID ultimately had a positive impact on the economic management of the hospital and patient’s safety.
