# Correction to: Implementation and Evaluation of a RFID Smart Cabinet to Improve Traceability and the Efficient Consumption of High Cost Medical Supplies in a Large Hospital

**DOI:** 10.1007/s10916-019-1492-1

**Published:** 2019-11-13

**Authors:** María del Carmen León-Araujo, Elisa Gómez-Inhiesto, María Teresa Acaiturri-Ayesta

**Affiliations:** 1 Purchasing and Repository Department, Cruces University Hospital, Barakaldo, Spain; 2 Departamento de Compras y Almacén, Hospital Universitario Cruces, Plaza de Cruces n° 12, 48903 Barakaldo, Bizkaia Spain

**Correction to: Journal of Medical Systems (2019) 43: 178**

10.1007/s10916-019-1269-6

The article Implementation and Evaluation of a RFID Smart Cabinet to Improve Traceability and the Efficient Consumption of High Cost Medical Supplies in a Large Hospital, written by María del Carmen León-Araujo, Elisa Gómez-Inhiesto and María Teresa Acaiturri-Ayesta was originally published Online First without Open Access. After publication in volume 43, issue 6, page 178 the author decided to opt for Open Choice and to make the article an Open Access publication. Therefore, the copyright of the article has been changed to © The Author(s) 2019 and the article is forthwith distributed under the terms of the Creative Commons Attribution 4.0 International License (http://creativecommons.org/licenses/by/4.0/), which permits use, duplication, adaptation, distribution and reproduction in any medium or format, as long as you give appropriate credit to the original author(s) and the source, provide a link to the Creative Commons license, and indicate if changes were made.

